# Symptomatic Non-stenotic Carotid Disease in Embolic Stroke of Undetermined Source: Analysis of the ESCAPE-NA1 Trial

**DOI:** 10.1007/s00062-023-01365-0

**Published:** 2023-12-18

**Authors:** Martha Marko, Nishita Singh, Johanna M. Ospel, Kazutaka Uchida, Mohammed A. Almekhlafi, Andrew M. Demchuk, Raul G. Nogueira, Ryan A. McTaggart, Alexandre Y. Poppe, Jeremy L. Rempel, Michael Tymianski, Michael D. Hill, Mayank Goyal, Bijoy K. Menon, the ESCAPE-NA1 Investigators

**Affiliations:** 1 Department of Neurology, Medical University of Vienna, Waehringer Guertel 18–20, 1090 Vienna, Austria; 2 Department of Clinical Neurosciences, Cumming School of Medicine, University of Calgary, Calgary, AB Canada; 3 Department of Diagnostic Imaging, Cumming School of Medicine, University of Calgary, Calgary, AB Canada; 4 Department of Neurosurgery, Hyogo Medical University, Nishinomiya, Japan; 5 Hotchkiss Brain Institute, Cumming School of Medicine, University of Calgary, Calgary, AB Canada; 6 Department of Community Health Sciences, Cumming School of Medicine, University of Calgary, Calgary, AB Canada; 7 Emory University School of Medicine, Grady Memorial Hospital, Atlanta, USA; 8 Warren Alpert School of Medicine, Brown University, Providence, RI USA; 9 Department of Medicine (Neurology), Centre Hospitalier de l’Université de Montréal, Montreal, QC Canada; 10 University of Alberta Hospital, Edmonton, AB Canada; 11 NoNO, Toronto, ON Canada; 12 Department of Medicine, Cumming School of Medicine, University of Calgary, Calgary, Canada

**Keywords:** Ischemic stroke, Stroke etiology, Carotid stenosis, Computed tomography angiography, Atherosclerotic plaque

## Abstract

**Purpose:**

Non-stenotic (< 50%) carotid disease may play an important etiological role in ischemic stroke classified as embolic stroke of undetermined source (ESUS). We aimed to assess the prevalence of non-stenotic carotid disease and its association with ipsilateral ischemic stroke.

**Methods:**

Data are from ESCAPE-NA1, a randomized controlled trial investigating the neuroprotectant nerinetide in patients with acute ischemic stroke and large vessel occlusion (LVO). The degree of stenosis of the extracranial internal carotid artery (ICA) and high-risk plaque features were assessed on baseline computed tomography (CT) angiography. We evaluated the association of non-stenotic carotid disease and ipsilateral stroke by age-adjusted and sex-adjusted logistic regression and calculated the attributable risk of ipsilateral stroke caused by non-stenotic carotid disease.

**Results:**

After excluding patients with non-assessable imaging, symptomatic > 50% carotid stenosis and extracranial dissection, 799/1105 (72.1%) patients enrolled in ESCAPE-NA1 remained for this analysis. Of these, 127 (15.9%) were classified as ESUS. Non-stenotic carotid disease occurred in 34/127 ESUS patients (26.8%) and was associated with the presence of ipsilateral ischemic stroke (odds ratio, OR 1.6, 95% confidence interval, CI 1.0–2.6, *p* = 0.049). The risk of ipsilateral ischemic stroke attributable to non-stenotic carotid disease in ESUS was estimated to be 19.7% (95% CI −5.7% to 39%), the population attributable risk was calculated as 4.3%. Imaging features such as plaque thickness, plaque irregularity or plaque ulceration were not different between non-stenotic carotids with vs. without ipsilateral stroke.

**Conclusion:**

Non-stenotic carotid disease frequently occurs in patients classified as ESUS and is associated with ipsilateral ischemic stroke. Our findings support the role of non-stenotic carotid disease as stroke etiology in ESUS, but further prospective research is needed to prove a causal relationship.

**Supplementary Information:**

The online version of this article (10.1007/s00062-023-01365-0) contains supplementary material, which is available to authorized users.

## Introduction

Embolic stroke of undetermined source (ESUS) accounts for up to 25% of all ischemic strokes [1]. Various potential thromboembolic sources, including cardiac valvular abnormalities, covert atrial fibrillation, paradoxical embolism or arterio-arterial emboli fall under the ESUS rubric [2]. Using the TOAST criteria, large artery atherosclerosis (LAA) as the cause of stroke only applies if there is more than 50% luminal narrowing of the ipsilateral internal carotid artery [3]; however, non-stenotic carotid disease, defined as < 50% luminal narrowing of the internal carotid artery, can be an underlying source of emboli and cause of ischemic stroke and yet these patients are still classified as cryptogenic stroke or ESUS [4]. A systematic review showed a prevalence of non-stenotic carotid disease of 51% in patients with ischemic stroke [5]. Further meta-analyses of patients with non-stenotic carotid disease showed a first-ever risk of stroke or transient ischemic attack (TIA) of 0.5/100 person-years and a recurrence risk of ischemic events of 2.6/100 person-years [6]. This risk may further increase in the presence of high-risk plaque features, such as plaque ulceration, intraplaque hemorrhage or increased plaque thickness [7]. Based on these findings, there has been a recent suggestion to classify these non-stenotic plaques into a possible, probable, and definite source of ischemic stroke (symptomatic non-stenotic carotid plaque, SyNC) to increase awareness of their contribution to stroke etiology [8].

Hypothesizing a relevant role of non-stenotic carotid disease in stroke etiology, we aimed to assess the prevalence of non-stenotic carotid disease in the ESCAPE-NA1 patient sample focusing on patients classified as ESUS. We furthermore aimed to evaluate the association between non-stenotic carotid disease and risk of ipsilateral ischemic stroke and to investigate the influence of high-risk plaque features on computed tomography angiography (CTA) on the occurrence of ischemic stroke in patients with ESUS.

## Methods

### Study Population

ESCAPE-NA1 is a multicenter, international, randomized controlled trial investigating the effect of the intravenous neuroprotectant nerinetide vs. placebo in patients with acute ischemic stroke (AIS) and large vessel occlusion (LVO) presenting within 12 h of symptom onset. Detailed inclusion and exclusion criteria as well as study procedures have been published previously [9]. Stroke etiology was determined by the site investigator as either cardioembolic, large artery atherosclerosis (LAA) with extracranial stenosis, LAA with extracranial dissection, LAA with intracranial stenosis, ESUS or undetermined etiology. Ethics approval was provided by the local ethics boards; patients or their legal representatives provided informed consent [9].

**Fig. 1:**
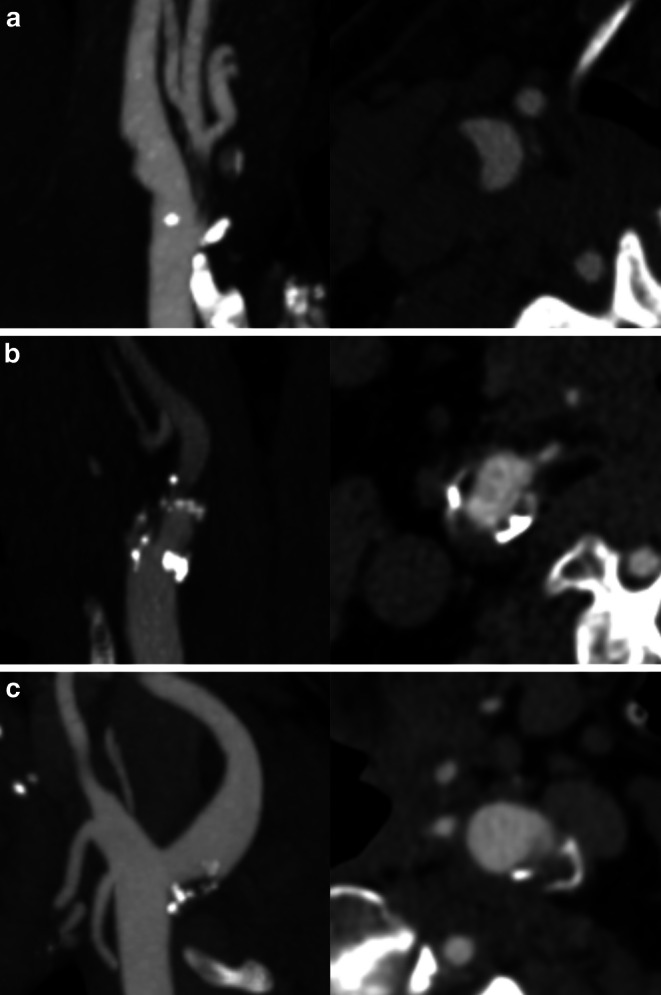
Sagittal reformatted (*left*) and axial (*right*) images of three exemplary cases (**a**–**c**) of extracranial internal carotid arteries with non-stenotic disease

For this sub-analysis, we stratified patients according to stroke etiology into patients classified as ESUS and non-ESUS. Patients with symptomatic carotid stenosis (defined as > 50% stenosis of the extracranial carotid artery and an ipsilateral ischemic stroke), patients with extracranial carotid dissection and patients with nonassessable extracranial carotid arteries due to limited imaging availability were excluded (see patient flowchart, Online resource—Fig. 1).

### Imaging Assessment

The baseline head and neck CT-angiogram was reviewed by two stroke neurologists (MM, NS) by consensus. Conflicts were resolved by a senior neuroradiologist (MG). The degree of carotid stenosis was assessed on axial source images and sagittal reformations and scored according to the NASCET criteria [10]. The inner diameter of the carotid lumen was measured at the narrowest point of the proximal internal carotid artery and 1–2 cm distal to the carotid bulb at a point where vessel walls were parallel [11]. Plaque assessment including evaluation of the presence of high-risk plaque features (plaque irregularity, plaque ulceration, plaque thickness, presence of a carotid web, and plaque calcification) was performed in line with previously published methodology [12]. Non-stenotic carotid disease was defined as one or more plaque in the cervical segment of the internal carotid artery causing < 50% luminal narrowing. Exemplary cases for non-stenotic carotid disease are given in Fig. 1. Predominantly calcified plaques (defined as ≥ 50% calcified plaque components on visual inspection) were excluded from this definition due to previously published data suggesting a decreased risk of ischemic stroke in these patients [13]. The primary outcome parameter was ischemic stroke which was scored according to the side of the intracranial vessel occlusion at baseline. As per definition of the study inclusion criteria in the ESCAPE-NA1 trial, only subjects with proximal vessel occlusions in the anterior circulation (intracranial ICA, M1 or functional M1, all M2 branches occluded) were included [9].

### Statistical Analysis

The prevalence of non-stenotic carotid disease at a patient level (i.e., the prevalence of unilateral or bilateral non-stenotic carotid disease in a patient) and of non-stenotic carotid disease associated with ipsilateral ischemic stroke is reported using standard descriptive statistics. Baseline characteristics (demographic, clinical and imaging variables) were then compared amongst patients with ESUS and patients with non-ESUS as well as patients with non-stenotic carotid disease vs. patients without non-stenotic carotid disease in the ESUS subgroup using descriptive statistics (t-test, Wilcoxon rank-sum test and Fisher’s exact test as applicable). Plaque characteristics (degree of stenosis 0%, 1–29%, 30–49%, plaque thickness dichotomized to </≥ 3 mm, plaque irregularity, plaque ulceration, presence of a carotid web, focal caliber increase and the combination of high-risk plaque features) were assessed for each carotid artery in a patient and compared between carotids with symptomatic non-stenotic disease (i.e., patients with non-stenotic carotid disease and ipsilateral stroke) and patients with non-stenotic carotid disease but no ipsilateral stroke. Logistic regression modelling adjusted for age and sex was performed to evaluate the association of non-stenotic carotid disease with ipsilateral ischemic stroke at a carotid level; patient-ID was therefore included as random effects variable in such analysis. The risk of ipsilateral stroke attributable to the non-stenotic carotid disease (attributable risk, AR) was calculated by estimating the risk ratio (RR) using a generalized linear model with log link for the association between non-stenotic carotid disease and ipsilateral stroke and using the formula AR = RR − 1 /RR. In addition, the population attributable risk of symptomatic non-stenotic carotid disease was calculated according to the formula AF_p_ = [P_e_(RR-1)]/[1+P_e_(RR-1)], where AF_p_ is the population attributable fraction and P_e_ is the exposed population [14]. P‑values < 0.05 were considered statistically significant. All analyses were performed using STATA, version 16.0 (StataCorp LLC, College Station, TX, USA).

## Results

A total of 1105 patients were enrolled in the ESCAPE-NA1-trial. Of these 141 were classified as ESUS and of these, 14 had non-assessable carotid imaging, leaving 127 ESUS patients. After excluding patients with ipsilateral > 50% carotid stenosis/occlusion (*n* = 220) or carotid dissection and ipsilateral stroke (*n* = 20), and non-assessable carotid imaging (*n* = 52), there were 672 non-ESUS patients for clinical comparison. In comparison to non-ESUS patients, patients with ESUS were younger, less likely to have hypertension but more likely to have a history of smoking and were treated with intravenous thrombolysis more frequently (Online resource—Table 1). There was no difference in the prevalence of non-stenotic carotid disease in patients classified as ESUS compared to patients classified as non-ESUS (ESUS: 27.9% vs. non-ESUS 26.1%, *p* = 0.826; Online resource—Table 1).

In the ESUS group, 34 patients (26.8%) had extracranial non-stenotic carotid disease, 13 of whom (10.2% of the ESUS patients) presented with bilateral non-stenotic carotid disease. Baseline characteristics of ESUS patients with vs. without non-stenotic carotid disease are provided in Table 1. Overall, baseline characteristics were comparable between the two groups, except for age (median age, non-stenotic disease: 67.6 years vs. no non-stenotic carotid disease: 62.1 years, *p* = 0.030) and history of prior stroke/TIA (non-stenotic disease: 29.4% vs. no non-stenotic disease: 11.8%, *p* = 0.029). When analyzing the same data at a carotid level, i.e., 254 carotids from 127 patients with ESUS, 47 carotids (18.5%) had non-stenotic carotid disease. Of these, 28 carotids (59.6% of non-stenotic carotids, 11.0% of all carotids) presented with an ipsilateral ischemic stroke. Imaging features such as plaque thickness, plaque irregularity or plaque ulceration were not different between non-stenotic carotids with vs. without ipsilateral stroke (Table 2).

**Table 1:** Baseline characteristics for ESUS patients with non-stenotic carotid disease vs. ESUS patients without non-stenotic carotid disease

	ESUS patients with non-stenotic carotid disease (*n* = 34)	ESUS patients without non-stenotic carotid disease (*n* = 93)	*p*-value
*Age, years, median (IQR)*	67.6 (61.0–77.8)	62.1 (52.7–72.6)	**0.030**
*Female sex, n (%)*	17 (50.0)	45 (48.4)	1.000
*Baseline NIHSS, median (IQR)*	18 (14–23)	17 (12–21)	0.191
**Medical history**			
*Hypertension, n (%)*	23 (67.7)	48 (51.6)	0.157
*Diabetes, n (%)*	9 (26.5)	14 (15.1)	0.121
*Prior stroke/TIA, n (%)*	10 (29.4)	11 (11.8)	**0.029**
*Recent stroke/TIA, n (%)*	1 (2.9)	7 (7.5)	0.681
*Hyperlipidemia, n (%)*	17 (50.0)	35 (37.6)	0.227
*Smoking, n (%)*	18 (52.9)	56 (60.9)	0.541
**Imaging characteristics**			
*Baseline ASPECTS, median (IQR)*	8 (7–9)	8 (7–9)	0.740
*Collaterals*			
Good, *n* (%)	4 (11.8)	15 (16.1)	0.160
Moderate, *n* (%)	30 (88.2)	70 (75.3)	
Poor, *n* (%)	0 (0)	8 (8.6)	
*Intracranial occlusion location*			
ICA, *n* (%)	3 (8.8)	15 (16.1)	0.601
M1, *n* (%)	29 (85.3)	74 (89.6)	
M2, *n* (%)	2 (5.9)	4 (4.3)	
*Intravenous thrombolysis, n (%)*	24 (70.6)	70 (75.3)	0.650

Statistically significant results are marked in **bold**

*ESUS* embolic stroke of undetermined source, *NIHSS* National Institutes of Health Stroke Scale, *mRS* modified Rankin scale, *TIA* transient ischemic attack, *ASPECTS* Alberta Stroke Program Early CT Score, *ICA* internal carotid artery, *IQR* interquartile range

**Table 2:** Plaque features in non-stenotic carotid arteries with vs. without ipsilateral ischemic stroke in ESUS patients

	Ipsilateral stroke (*n* = 28)	No ipsilateral stroke (*n* = 19)	*p*-value
*Plaque thickness dichotomized, >* *3* *mm n (%)*	8 (28.6)	4 (21.1)	0.737
*Irregular plaque, n (%)*	7 (25.0)	4 (21.1)	1.000
*Ulcerated plaque, n (%)*	2 (7.1)	1 (5.3)	1.000
*Degree of stenosis*			
0%	2 (7.1)^b^	0 (0)	0.727
1–29%	21 (75.0)	16 (84.2)	
30–49%	5 (17.9)	3 (15.8)	
*Carotid web, n (%)*	3 (10.7)	0 (0)	0.262
*>* *1 high-risk feature*^*a*^*, n (%)*	6 (21.4)	3 (15.8)	0.720

*ESUS* embolic stroke of undetermined source

^a^High-risk plaque features: > 29% stenosis, irregular plaque, ulcerated plaque, ≥ 3 mm plaque thickness

^b^Two patients presented with a carotid web but an otherwise unremarkable carotid and are therefore labelled as no stenosis but non-stenotic carotid disease

In logistic regression analysis adjusted for age and sex amongst ESUS patients, the presence of non-stenotic carotid disease was associated with ipsilateral stroke (adjusted OR 1.6, 95% CI 1.0–2.6, *p* = 0.049). The risk of ipsilateral ischemic stroke attributable to the presence of non-stenotic carotid disease was estimated to be 0.197 (95% CI −0.057 to 0.390) indicating that in the sample of ESUS patients with non-stenotic disease and ipsilateral stroke in this study, approximately 20% of ipsilateral ischemic lesions could be attributed to the non-stenotic internal carotid artery. The population attributable risk was calculated as 0.043, indicating that in a population of ESUS patients 4.3% of ischemic events can be attributed to symptomatic non-stenotic carotid disease.

## Discussion

In our study population of patients with large vessel ischemic stroke classified as ESUS we observed an overall prevalence of non-stenotic carotid disease of 26.8%. In addition, we could show a significant association between the presence of non-stenotic carotid disease and ipsilateral ischemic stroke.

The reported prevalence of non-stenotic carotid disease in patients with ESUS in these data from the ESCAPE-NA1 trial lies within the range of previously reported findings. A substudy from the INTERRSeCT trial reported a prevalence of 39.1% in an ESUS population and was also able to show a significant association between non-stenotic carotid disease and ipsilateral stroke [12]. Similarly, a secondary analysis from the STRATIS registry reported a prevalence of 22.7% in the ESUS subgroup and a significant association with ipsilateral ischemic stroke. As in our study, the STRATIS registry also consisted of patients with LVO who received endovascular treatment (EVT) [15]. These results suggest that a substantial subgroup of patients classified as having ESUS may potentially have ischemic stroke as a result of ipsilateral non-stenotic carotid disease. Non-stenotic carotid disease has also been shown to be present in non-ESUS patients, such as patients presumed to have a cardioembolic source of stroke. Non-stenotic carotid disease could be a competing etiology in many such patients [16]. Overall, our results provide further evidentiary support for non-stenotic carotid disease as a relevant stroke etiology, especially in the subgroup of patients currently classified as embolic stroke of undetermined source.

A number of patients in our sample presented with bilateral non-stenotic carotid disease. The reported frequency of bilateral non-stenotic carotid disease of 10.2% in ESUS patients in our analyses is similar to previous analyses. In comparison, 8.7% and 9.2% of ESUS patients presented with bilateral non-stenotic disease in the secondary INTERRSeCT and STRATIS analyses, respectively [12, 15]. In these patients, non-stenotic carotid disease might be a part of systemic atherosclerosis and a marker of increased overall cardiovascular risk [17, 18].

Previous studies have identified various high-risk plaque features associated with an increased risk of ipsilateral stroke in patients with non-stenotic carotid disease [7, 13]; however, we were unable to determine any relevant imaging features that might discriminate between the non-stenotic carotid that is associated with ipsilateral stroke and that which is not (Table 2). Interestingly, a combination of more than one plaque feature was numerically slightly more frequent in non-stenotic carotid disease with ipsilateral stroke without reaching statistical significance. Moreover, the prevalence of high-risk plaque features was low in our patient sample and plaque feature assessment was based on CT imaging. Previous studies investigating high-risk plaque features in carotid stenosis predominantly assessed those characteristics on MRI which enable a more detailed assessment of specific plaque characteristics. A more detailed plaque analysis and characterization may be helpful for further risk stratification [19–21].

Our study has several limitations. Firstly, our study population was restricted to patients with LVOs which might constitute a selection bias and limits the generalizability of our results to an overall population of patients with ischemic stroke. Secondly, our analysis is based on a retrospective assessment of CT imaging of a randomized controlled trial, which was not designed to investigate the role of non-stenotic carotid disease in stroke etiology. A prospectively followed population combining both large vessel and non-large vessel occlusion strokes to assess the prevalence of non-stenotic carotid disease and the risk of ipsilateral stroke would be a recommendable study set-up to further investigate this condition. Third, stroke etiology was classified by the site investigator and not by central adjudication, limiting the available information on etiological work-up and diagnosis of ESUS. Last, the prevalence of ESUS in our patient sample was small and the analysis therefore likely underpowered with limited generalizability to larger ESUS populations. In addition, this might especially affect the analysis of associations of specific plaque features in non-stenotic disease with ipsilateral stroke and might have influenced the borderline significance of some of the presented results. These results, therefore, need to be interpreted cautiously even though they are in line with previously reported analyses.

In conclusion, a substantial number of patients with ischemic stroke classified as embolic stroke of undetermined source (ESUS) present with non-stenotic (< 50% stenosis) carotid disease. In these patients, non-stenotic carotid disease is associated with ipsilateral ischemic stroke suggesting a potential role as stroke etiology. Prospective studies investigating the association of non-stenotic carotid disease and ischemic stroke are needed to better determine if a causal relationship exists between these two conditions. In addition, increased awareness of non-stenotic carotid disease as a potential stroke etiology especially in patients with ESUS and additional diagnostic work-up including dedicated vessel imaging could facilitate improvements in stroke care for these patients.

### Supplementary Information

Online Resource

## Data Availability

N/A
