# Supplementary material for: Symptomatic Non-stenotic Carotid Disease in Embolic Stroke of Undetermined Source: Analysis of the ESCAPE-NA1 Trial
Source: Clin Neuroradiol. 2023 Dec 18;34(2):333–9. doi: 10.1007/s00062-023-01365-0 (PMC11130033; doi:10.1007/s00062-023-01365-0)
Supplement: Supplementary file 1 — Online Resource [file 62_2023_1365_MOESM1_ESM.pdf]

SUPPLEMENTARY INFORMATION - ONLINE RESOURCE

SYMPTOMATIC NON-STENOTIC CAROTID DISEASE IN EMBOLIC STROKE OF UNDETERMINED SOURCE – ANALYSIS OF THE ESCAPE-NA1 TRIAL

FIGURES:

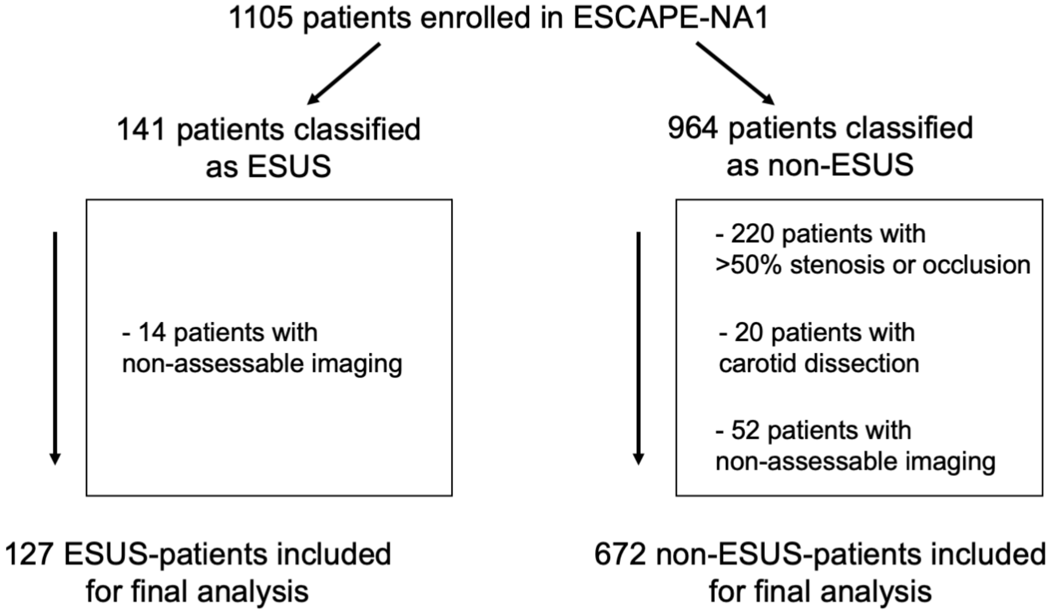

Online Resource – Figure 1: Patient flowchart

TABLES

**Online Resource - Table 1**: Baseline characteristics for patients with ESUS compared to patients with Non-ESUS			
	ESUS-Patients (n = 127)	Non-ESUS Patients (n = 672)	p-Value
Age, median (IQR)	63.9 (54.7-74.3)	73.8 (63.3-81.8)	**<0.001**
Female sex, n (%)	62 (48.8)	373 (54.3)	0.175
Baseline NIHSS, median (IQR)	17 (13-21)	17 (12-21)	0.935
Medical history			
Hypertension, n (%)	71 (55.9)	503 (74.9)	**<0.001**
Diabetes, n (%)	23 (18.1)	142 (21.1)	0.819
Prior stroke/TIA, n (%)	21 (16.5)	98 (14.6)	0.587
Recent stroke/TIA, n (%)	8 (6.3)	20 (3.0)	0.069
Hyperlipidemia, n (%)	52 (41.0)	327 (48.7)	0.121
Smoking, n (%)	74 (58.7)	287 (42.9)	**0.001**
Imaging characteristics			
Baseline ASPECTS, median (IQR)	8 (7-9)	8 (7-9)	0.619
Collaterals			
Good, n (%)	19 (15.0)	113 (17.0)	0.372
Moderate, n (%)	100 (78.7)	530 (79.3)	
Poor, n (%)	8 (6.3)	25 (3.7)	
Intracranial occlusion location			
ICA, n (%)	18 (14.2)	128 (19.2)	0.271
M1, n (%)	103 (81.1)	519 (77.8)	
M2, n (%)	6 (4.7)	20 (3.0)	
Non-stenotic carotid disease, n (%)	34 (27.0)	174 (26.1)	0.826
Treatment			
Intravenous thrombolysis, n (%)	94 (74.0)	376 (56.0)	**<0.001**
Nerinetide, n (%)	54 (42.5)	332 (49.4)	0.175

ESUS indicates Embolic Stroke of Undetermined Source, NIHSS National Institute of Health Stroke Scale, mRS modified Rankin Scale, TIA Transient Ischemic Attack, ASPECTS Alberta Stroke Program Early CT Score, ICA Internal Carotid Artery
